# Spatiotemporal and Transcriptional Characterization on Tanshinone Initial Synthesis in *Salvia miltiorrhiza* Roots

**DOI:** 10.3390/ijms232113607

**Published:** 2022-11-06

**Authors:** Caicai Lin, Lin Zhang, Xia Zhang, Xin Wang, Chaoyang Wang, Yufeng Zhang, Jianhua Wang, Xingfeng Li, Zhenqiao Song

**Affiliations:** 1 Agronomy College, Shandong Agricultural University, Tai’an 271018, China; 2 State Key Laboratory of Crop Biology, Shandong Agricultural University, Tai’an 271018, China

**Keywords:** *Salvia miltiorrhiza*, tanshinone, full-length transcriptome, Oxford nanopore technology

## Abstract

Tanshinones are the bioactive constituents of Danshen (*Salvia miltiorrhiza* Bunge), which is used in Traditional Chinese Medicine to treat cardiovascular and other diseases, and they synthesize and accumulate in the root periderm of *S. miltiorrhiza*. However, there is no relevant report on the initial stage of tanshinone synthesis, as well as the root structure and gene expression characteristics. The present study aims to provide new insights into how these bioactive principles begin to synthesize by characterizing possible differences in their biosynthesis and accumulation during early root development from both spatial and temporal aspects. The morphological characteristics and the content of tanshinones in roots of *S. miltiorrhiza* were investigated in detail by monitoring the seedlings within 65 days after germination (DAGs). The ONT transcriptome sequencing was applied to investigate gene expression patterns. The periderm of the *S. miltiorrhiza* storage taproot initially synthesized tanshinone on about 30 DAGs. Three critical stages of tanshinone synthesis were preliminarily determined: preparation, the initial synthesis, and the continuous rapid synthesis. The difference of taproots in the first two stages was the smallest, and the differentially expressed genes (DEGs) were mainly enriched in terpene synthesis. Most genes involved in tanshinone synthesis were up regulated during the gradual formation of the red taproot. Plant hormone signal transduction and ABC transport pathways were widely involved in *S. miltiorrhiza* taproot development. Five candidate genes that may participate in or regulate tanshinone synthesis were screened according to the co-expression pattern. Moreover, photosynthetic ferredoxin (FD), cytochrome P450 reductase (CPR), and CCAAT binding transcription factor (CBF) were predicted to interact with the known downstream essential enzyme genes directly. The above results provide a necessary basis for analyzing the initial synthesis and regulation mechanism of Tanshinones.

## 1. Introduction

*Salvia miltiorrhiza* is a well-known perennial herb plant of the genus *Salvia*, Lamiaceae family. Its red storage roots are known as ‘Danshen’, a Traditional Chinese Medicine (TCM) used for over 2000 years. Pharmacological studies have shown that *S. miltiorrhiza* have a variety of biological activities, such as anti-arrhythmia, anti-atherosclerosis, anti-inflammation, and anti-oxidation [1,2,3,4]. In addition, *S. miltiorrhiza* has been considered an ideal model species for TCM [5,6].

The characteristic orange–red pigment in Danshen is mainly composed of numerous nor-diterpenoid tanshinones, some of them being primary bioactive ingredients, such as cryptotanshinone, tanshinone I, tanshinone IIA, and tanshinone IIB [7], which are mainly synthesized and accumulated in the periderm of the root of *S. miltiorrhiza* [8,9]. The redder the color in the root with a specific diameter, the higher the tanshinone content.

The synthetic pathway of tanshinones has not been thoroughly analyzed [10]. Geranylgeranyl diphosphate (GGPP), a precursor of diterpenoids, was synthesized through the mevalonate pathway (MVA) located in the cytoplasm and the 2-methyl-D-erythritol-4-phosphate pathway (MEP) located in the plastid. The GGPP was cyclized by copalyl diphosphate synthase (CPS) and kaurene synthase-like (KSL) to obtain a diterpene skeleton. The miltiradiene was modified by several cytochrome P450 terminal oxygenases (CYP450s) to produce a tanshinone intermediate containing CYP76AH1 and CYP76AK1, CYP76AH3, CYP71D373, and CYP71D375 [11,12,13]. However, the later modification of the tanshinone skeleton was still complex and unknown. Studies have shown that the expression of some genes has temporal and spatial correlation, especially the genes involved in secondary metabolic pathways that are easier to co-express [14,15].

The synthesis and accumulation of secondary metabolites in plants are induced by environmental conditions [16]. The genes controlling the synthesis of related enzymes in secondary metabolic biosynthesis are expressed only under specific environmental stimulation or stress [17,18]. Tanshinones were biosynthesized on the roots from a specific developmental process [19]. Previous studies focused more on the specific mechanism of tissue expression [19,20], but there was insufficient research on the expression characteristics and regulation mechanism of development specificity, especially in the initial synthesis stage of tanshinone.

Transcriptome sequencing technology can obtain the expression information of all genes under specific conditions, which has become the leading technical means of co-expression analysis, which can effectively screen target genes [21]. At present, the third-generation sequencing technology represented by Oxford Nanopore and PacBio SMRT, with the advantages of ultra-long reads length, short sequencing cycle, and direct reading nucleic acid modification, has begun to successfully apply to the study of the full-length transcriptome in animals and plants [4,22,23,24].

This study selected the initial synthesis stage of tanshinone for our research. The morphological characteristics, taproot tissue structure, and tanshinone content of *S. miltiorrhiza* seedlings grown within 65 days were investigated. Moreover, we used full-length transcriptome sequencing to investigate the essential enzyme genes and regulatory genes involved in tanshinone initial biosynthesis and mine regulation mechanisms related to a particular tissue in the developmental stage.

## 2. Results

### 2.1. Developmental Stage of S. miltiorrhiza Seedlings and Tanshinone Content

**Figure 1:**
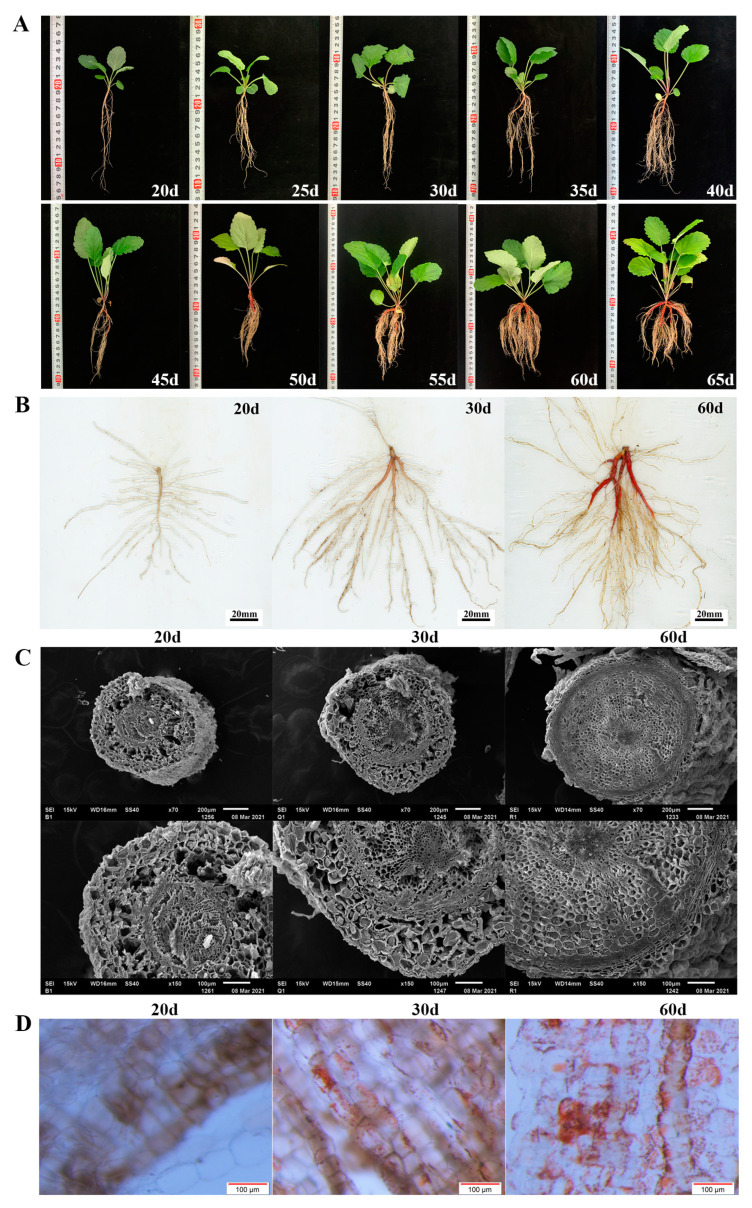
(**A**) Morphology of *S. miltiorrhiza* seedlings within 60 days of growth. (**B**) Scans of roots of *S. miltiorrhiza* seedlings grown for 20d (white root, B), 30d (light red root, Q), and 60d (red root, R). (**C**) The root cross-section of *S. miltiorrhiza* seedlings grown for 20, 30, and 60 days was observed under scanning electron microscope. (**D**) The root epidermis was observed under a stereomicroscope, and the magnification was 11.5×.

*S. miltiorrhiza* seedlings were investigated in detail within 15–65 days after germination (DAG) (Figure 1, Table 1). There was no accumulation of red matter in all hairy roots at any development stage, and only red matter could be seen on the primary roots.

Within 25 DAG, the seedlings grew slowly, and the fresh weight of the leaves and roots increased slightly (Figure 1A). The ratio of root to shoot was less than one fifth. Most seedlings tended to have only one taproot. During this period, the epidermis of the taproot was white and transparent (Figure 1B). At the same time, UPLC results showed that tanshinone IIA was not detected in the roots (Table 1, Figure S1C). After 30 days, the fresh weight of the seedlings gradually increased. The growth rate of the root increased more quickly so that the ratio of root to shoot increased to one third (Figure 1A). At this time, the epidermis of the taproot started to turn red, and a small amount of tanshinone IIA was detected in the root (Table 1, Figure S1D). This result means that tanshinone begins to be synthesized in the root epidermis (Figure 1B).

Since then, the root color continued to deepen, and the content of tanshinone IIA also increased significantly. At 45 DAG, the taproot showed a bright brick red color, followed by the lateral root. At 60 DAG, the epidermis of the taproot and multiple lateral roots reached red (Figure 1), and the ratio of root to shoot increased to half. The consistency between the red degree of the root and tanshinone content was proved (Table 1, Figure S1E).

### 2.2. Root Structure by Scanning Electron Microscope and Stereomicroscope

We collected the taproots of *S. miltiorrhiza* seedlings growing for 20, 30, and 60 DAG and observed the differences in root structure with a scanning electron microscope and stereomicroscope simultaneously (Figure 1C,D). The results showed that red substances were not observed in the epidermal cells of the roots grown for 20 DAG, and only a tiny amount of red substances were found in roots grown for 30 DAG.

The observation of the cross-section of the taproot with a scanning electron microscope showed that the root growing for 20 DAG was a primary root, with a large number of cortical parenchyma cells with a loose arrangement and apparent intercellular space, accounting for a large proportion of the whole root. At 30 DAG, the cell volume increased, but the cells were still arranged loosely and had apparent intercellular space, similar to 20 DAG (Figure 1C).

At 60 DAG, a large number of red substances in roots was observed, and the cells were arranged closely and orderly and had no defects. The gradual thickening of the central column makes the peripheral skin break and fall off (Figure 1D), which indicates the rapid propagation and enrichment of phloem cells in the root system.

Therefore, we preliminarily determined three key growth stages: 25 days after germination was the preparation stage of tanshinone synthesis, 25–40 days (about 30 DAG) was the initial synthesis stage of tanshinone, and 40–65 days was the continuous and rapid synthesis stage of tanshinone.

### 2.3. Nanopore-Based RNA Sequencing and Quality Control

Given the significant difference in tanshinone IIA content, a total of nine samples from the root tissue of a *S.miltiorrhiza* seedling grown for 20 d (white roots, named B1, B2, and B3), 30 d (light red roots, named Q1, Q2, and Q3), and 60 d (red roots, named R1, R2, and R3) were collected to construct a full-length cDNA library. Then, the cDNA library was sequenced based on Oxford Nanopore Technologies (ONT) (Figure 1B). The clean data of each root sample produced by sequencing reached 2.07 GB (Table S2a). Full-length sequences were aligned with the reference genome to obtain consistent sequences with minmap2 software. The alignment efficiency of each sample exceeded 96.1% (the standard is ≥80%), indicating that the reference genome was consistent with the sequencing data. After removing redundant sequences, 31,566 transcripts were obtained (Table S2b).

### 2.4. Analysis of Differential Expression in Taproots of S. miltiorrhiza in Three Stages

**Figure 2:**
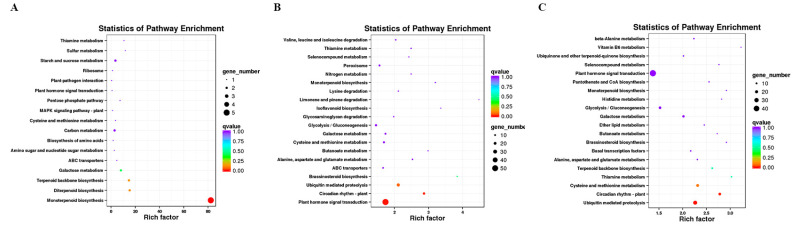
Scatter plot of KEGG pathway enrichment of up-regulated DEGs. (**A**) B vs. Q; (**B**) Q vs. R; (**C**) B vs. R.

In this study, DESeq2 was used for gene differential expression analysis between roots for 20, 30, and 60 days. Fold Change ≥1.5 and *p*-value < 0.01 were used as screening criteria, and 4377 differentially expressed genes were obtained, as shown in Table 2. Differential expression genes (DEGs) were annotated into KEGG, and the pathway enrichment analysis was carried out. The first 20 pathways with the lowest significance Q value were selected as the enrichment scatter diagram of the KEGG pathway of the differential expression transcript (Figure 2).

We identified 128 DEGs in the white root vs. light red root (B vs. Q) comparison, which was a minor difference in the three combinations, including 46 up-regulated DEGs and 82 down-regulated DEGs in the light red roots (Table 2). Among the DEGs, 38 up-regulated and 53 down-regulated DEGs were annotated by GO (Table S3a). The up-regulated DEGs were significantly enriched in GO terms such as cellular response to oxygen-containing compounds in BP, the integral component of a membrane in cell composition (CC), and catalytic activity in molecular function (MF). The monoterpenoid biosynthesis pathway was the most significantly enriched metabolic pathway, followed by diterpenoid biosynthesis, terpenoid backbone biosynthesis, galactose metabolism, and ABC transporters (Figure 2A). Most of the down-regulated DEGs were significantly enriched in the cellular response to lipids; cellular component biogenesis; integral component of membrane and catalytic activity; and nitrogen metabolism, stilbenoid, diarylheptanoid, and gingerol biosynthesis, and tyrosine metabolism (Figure S2A).

In the light red root vs. red root comparison (Q vs. R), 3247 DEGs contained 1365 up-regulated and 1882 down-regulated DEGs in the red roots, and 1027 up-regulated and 1549 down-regulated DEGs were significantly enriched by GO (Table S3b). The up-regulated DEGs were significantly enriched in response to hormone and cellular responses to oxygen-containing compounds in BP, the CCAAT binding factor complex and the integral component of the membrane in CC, and the catalytic activity and calcium binding in MF. A total of 437 up-regulated differential genes were annotated to 116 metabolic pathways. Plant hormone signal transmission, plant circadian rhythms, Ubiquitin-mediated protein, brassinosteroid biosynthesis, and monoterpenoid biosynthesis significantly enriched five metabolic pathways (Figure 2B). The down-regulated DEGs were significantly enriched in the ribosome, proteasome, and DNA replication pathways (Figure S2B).

The highest number of DEGs (3672) was identified in the white root vs. red root comparisons (B vs. R), including 1516 up-regulated DEGs and 2156 down-regulated DEGs in the red roots; among them, 1138 up-regulated DEGs and 1777 down-regulated differential genes were analyzed by GO enrichment (Table S3c). The enrichment of DEGs in BP, CC, and MF categories was similar to those between Q vs. R. The response to hormone, cellular response to oxygen-containing compounds, protein retention in ER lumen in BP, CCAAT binding factor complex in CC, and catalytic activity in MP were significantly enriched GO terms. A total of 479 up-regulated unigenes were significantly enriched in the top five metabolic pathways, including Ubiquitin-mediated protein, plant circadian rhythms, cysteine and methionine metabolism, thiamine metabolism, and terpenoid backbone biosynthesis (Figure 2C). Down-regulated genes were significantly enriched in GO terms such as translation, ribosome, and the structural constitution of ribosome. Besides the three metabolic pathways mentioned in Q vs. R, the enrichment of down-regulated genes had photosynthesis-antenna proteins (Figure S2C).

### 2.5. Regulation Pathways Identification

The response to hormones significantly enriched GO terms in B vs. R and Q vs. R. Combined with the results of GO annotation and KEGG analysis, we predicted that plant hormone signal transduction and the ABC transporters pathway might play an essential role in the development stage of seedling roots, and then we analyzed the differential genes enriched in this pathway. In the expression profiles of the white root vs. light red root comparison, the salicylic acid signal transduction pathway showed that the differential gene annotated PR-1 (EVM0023500) was up regulated in the light red root (Figure S3). In addition, the expression of five differential genes was down regulated in the light red root, including the B-ARR gene of two cytokine signal transduction pathways (EVM0011500; EVM0025243), the GID1 (EVM0018632) and DELLA (EVM0018968) gene of the gibberellin signal transduction pathway, and the TCH4 (EVM0021658) gene in the brassinolide signal transduction pathway. We speculated that the differential genes of these pathways might be related to the initial synthesis of tanshinone.

The expression of different genes in the plant hormone signal transduction pathway was much more complex in Q vs. R (Figure S4). Each hormone signaling pathway had annotated a large number of different genes. The expression trends of several types of genes located in the same signal pathway were quite different, and some unigenes annotated as the same type of genes also showed inconsistent expression trends. These differential genes showed complex expression patterns, which might be due to the complex life activities in the roots in the two developmental stages, such as root thickening, the accumulation of secondary metabolites, and the formation of lateral roots.

In the B vs. R comparison, the ABC transporters pathway was a significantly enriched metabolic pathway (Figure S5), and the differential gene (EVM0024942) annotated as the ATP binding cassette gene was member 1 (ABCB1) of the ABCB subfamily B (MDR/TAP). In Q vs. R, more differential genes were enriched into the ABC transporters pathway (Figure S6). In the ABCB subfamily, a total of six differential genes were annotated as ABCB1, of which four were up regulated in red roots, including EVM0024942, and the other two were down regulated in red roots. In addition, two up-regulated genes were annotated as ABCB9, and one up-regulated gene was annotated as ATM. ABCC1 in the ABCC subfamily, ABCD3 in the ABCD subfamily, and ABCG2 and SNQ2 in the ABCG subfamily were all down regulated in the red root. One ABCC10 gene of the ABCC subfamily and another SNQ2 gene of the ABCG subfamily were also up regulated. It can be seen that the ABC transporters pathway was also a complex regulation pathway from the light red root to red root development.

### 2.6. Expression of Genes Related to Tanshinone Synthesis

**Figure 3:**
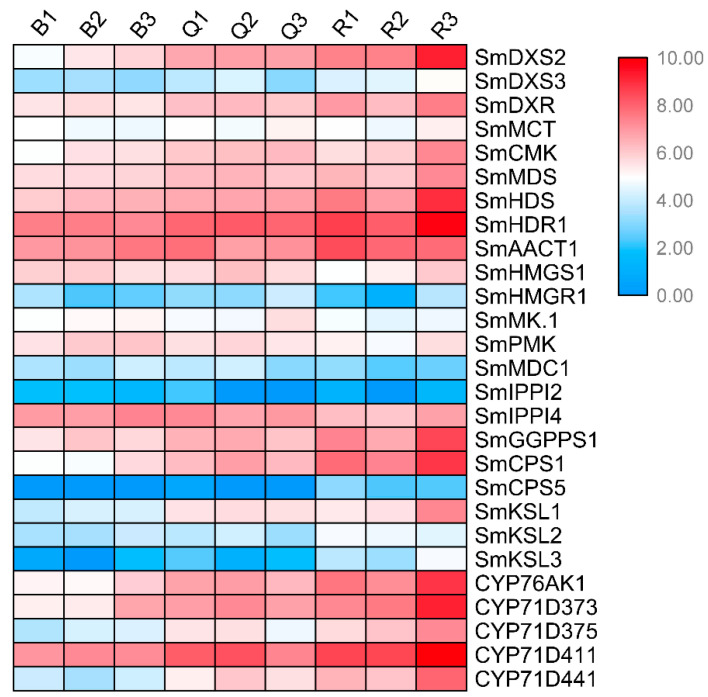
Expression patterns of known genes in tanshinone pathway in three kinds of roots.

We identified the expression patterns of all known genes of the tanshinone pathway at three developmental stages of the roots (Figure 3). It can be seen that the expression patterns of most genes were significantly higher in the red roots than in the light red roots, and they were the lowest in the white roots. In particular, the expression abundance of several essential enzyme genes in the downstream synthesis pathway was also high, such as *SmCPS1*, *SmKSL1*, *SmCYP76AK1*, *SmCYP71D373*, *SmCYP71D375*, and *SmCYP71D411*. These results showed that the expression of essential enzyme genes has a specific time and tissue specificity. At 30 days after seed germination, when the secondary structure began to appear in the root tissue, the expression of essential enzyme genes began to increase, indicating the initial synthesis of tanshinone, and the epidermal color of the root changed from white and transparent to light red. With the increase in time, the expression of these essential enzyme genes continued to be up regulated, resulting in the continuous accumulation of tanshinones in the pericarp, and the color of the root gradually deepened.

### 2.7. Screening Candidate Genes Based on Co-Expression Patterns

**Figure 4:**
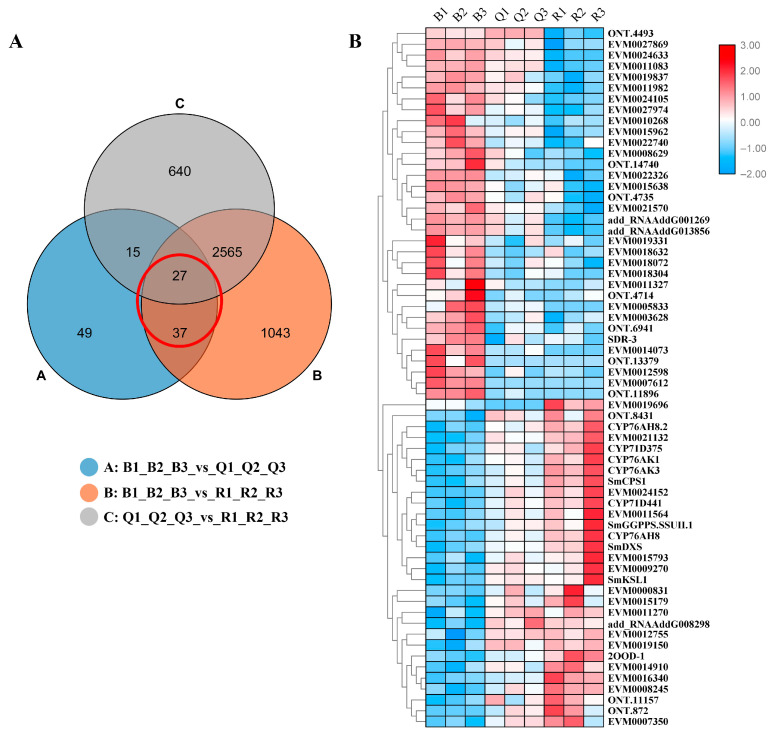
(**A**) Venn diagram of differentially expressed genes. Each circle in the figure represented a differential group, and the intersection of circles represented common differentially expressed genes. The corresponding number of genes was shown in the set. The adjacent genes have similar expression patterns in the (**B**) cluster diagram of 64 DEGs.

Many functionally related genes have very similar expression profiles under related conditions, mainly involved in the same synthetic pathway [9]. In order to further explore the candidate genes that may be involved in tanshinone biosynthesis, we analyzed all the differential genes. Based on the significant difference in tanshinone content, we focused on the common DEGs both in B vs. Q and B vs. R (Figure 4A). A total of 64 DEGs were screened.

**Figure 5:**
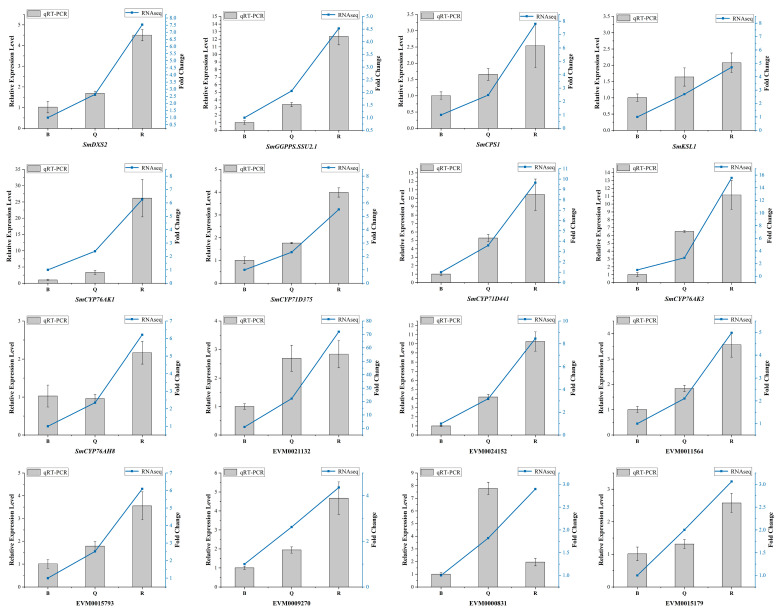
The qRT-PCR analysis of DEGs selected by transcriptome data. The standard deviation was represented by the error bars.

A cluster analysis showed that almost all differential genes were divided into two groups. Interestingly, five known essential enzyme genes in tanshinone synthesis were found in one group, including *SmDXS2*, *SmCPS1*, *SmKSL1*, *SmCYP76AK1*, and *SmCYP71D375* [25,26]. Several unknown genes had similar expression patterns with the known essential enzyme genes of the tanshinone synthesis pathway (Figure 4B). In order to confirm the accuracy of RNA-Seq, we verified the expression of six unknown genes and eight known genes with qRT-PCR (Figure 5). Moreover, the qRT-PCR analysis showed that five DEGs were explicitly expressed in the root tissue. Their expression level was significantly higher than that in the stem, leaf, and flower tissue, including EVM0011564, EVM0015793, EVM0015179, EVM0021132, and EVM0024152 (Figure S7). EVM0011564 was predicted to be a cytosolic factor and phosphatidylinositol transfer protein. EVM0015793 was annotated as SAM-dependent carboxyl methyltransferase and farnesoic acid carboxyl-o-methyltransferase, which may play a role in methyltransferase activity. EVM0021132 was annotated as a Kup system potassium uptake protein and transporter, which may be related to vital activities such as potassium ion transmembrane transporter activity and the integral component of the membrane. EVM0024152 was predicted to be a member of the terpene synthase family in *S. miltiorrhiza*, which was involved in the synthesis of terpenes; EVM0015179 was predicted to be alpha-glucosidase. Our results suggest that these genes have similar expression patterns with the known essential enzyme genes of the tanshinone biosynthesis pathway and may directly participate in or regulate the biosynthesis of tanshinone components. Their specific functions will be verified in future studies.

### 2.8. Prediction of the Protein–Protein Interactions (PPIs) Network of Differential Genes

In order to predict the interaction between the identified DEGs, we predicted the protein–protein interactions (PPIs) network of differential genes using the online string website. In the white root vs. light red root comparison, a total of seven differential genes were predicted to interact. They were annotated as calcium-binding protein, RING-H2 zinc finger protein, and Verticillium wilt resistance-like protein, which were speculated to be related to the interaction of pathogens (Figure S8).

**Figure 6:**
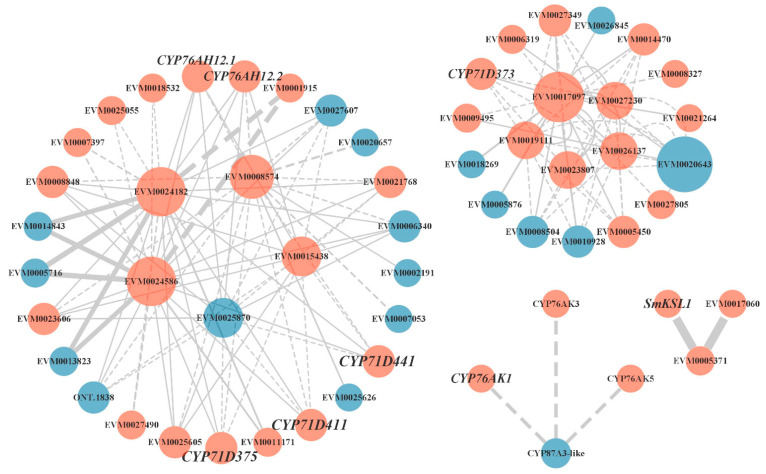
Protein–protein interaction network. Orange indicates that gene expression was up regulated in the red root, and blue indicates that gene expression was down regulated in red root.

Many differential proteins were predicted to interact in the light red root vs. red root and white root vs. red root comparisons. We focused on the protein–protein interaction of key enzyme genes in the tanshinone synthesis pathway (Figure 6). We screened the interaction proteins of several key enzyme proteins in the downstream pathway, including *SmKSL1*, *SmCYP76AK1*, *SmCYP71D373*, *SmCYP71D375*, and *SmCYP71D411*. Interestingly, *SmGGPPS*, *SmCYP71D375*, and *SmCYP71D411* may interact with five differential genes, including two genes encoding photosynthetic ferredoxin (FD) (EVM0015438, EVM0025870), one cytochrome P450 reductase (EVM0008574), and two CCAAT binding transcription factors (CBF-B/NF-YA) (EVM0024182, EVM0024586). Except for EVM0025870, the other four genes were up regulated in the light red root and a red root. Some CYPs genes were also predicted to interact with these five genes. *SmCYP71D373* interacted with one CCAAT binding transcription factor (CBF-B/NF-YA) (EVM0017097) and four CYP450 proteins, including CYP72A219 (EVM0019111), CYP72A327 (EVM0023807), CYP72A330 (EVM0026137), and CYP72A329 (EVM0027230). *SmKSL1* was predicted to interact with two other ent-kaurene synthases, and *SmCYP76AK1* was predicted to interact with CYP76AK2 and CYP76AK5. These results suggest that the activities of these predicted interacting proteins may have a certain impact on the synthesis of tanshinone.

## 3. Discussion

Tanshinones are the critical characteristic and active components extracted from the dry root and rhizome of *S. miltiorrhiza*, which is used in Traditional Chinese Medicine [27,28,29]. The root of *S. miltiorrhiza* is the central tissue of tanshinone biosynthesis and accumulation [8]. Presently, significant progress has been made in understanding tanshinone biosynthesis, and more genes involved in the synthesis pathway have been confirmed [8,15]. However, the regulation network and the biosynthetic pathway of final products such as tanshinone ⅡA have not been fully clarified.

At present, there have been some reports on the biosynthesis of tanshinone in the root of *S. miltiorrhiza* [8,9,30]. However, most of the previous studies used mature roots of *S. miltiorrhiza* as the plant materials. There are few studies on the temporal evaluation of the root growth and development of *S. miltiorrhiza* [31]. Therefore, we chose young roots of *S. miltiorrhiza* as the research material to explore the initial synthesis of tanshinone. In the present study, we preliminarily established the stage of tanshinone synthesis. Our results showed that tanshinone began to be detected in the taproot about 30 days after germination, and then it accumulated rapidly in several weeks. At the same time, the taproots underwent rapid and complex development. In previous studies, it was also shown that the root of *S. miltiorrhiza*, when grown for longer than 60 days, can accumulate a large amount of tanshinone [31].

Among B vs. Q, B vs. R, and Q vs. R, the morphological and structure difference between the white root and light red root (B vs. Q) was small, and so was the least DEG number. Previous studies demonstrated the localization of tanshinone to the root periderm [8,9,32]. The researchers observed the localization of tanshinone to the root periderm of three-year-old *S. miltiorrhiza* plants by phytochemical analysis and speculate that tanshinone biosynthesis may be completely carried out in the periderm [9]. Moreover, recent studies demonstrated that tanshinones were only distributed in the root periderm through a MALDI mass spectrometry imaging experiment, and the authors speculated that tanshinone could be synthesized and accumulated in situ in the root periderm, which may play an important role in protecting root tissue [8,32]. We therefore speculate that the formation of periderm tissue may be accompanied by the initial synthesis of tanshinone, and the tanshinone synthesis of root epidermal cells might be earlier than the propagation and enrichment of phloem cells in the root system.

**Figure 7:**
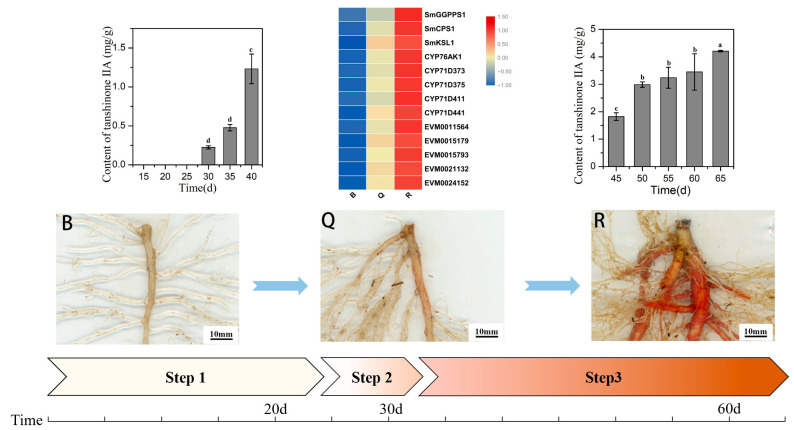
Schematic diagram of the initial synthesis process of tanshinone. B represent the white root, Q represent the light red root, R represent the red root, Step 1 represent the preparation stage, Step 2 represent the initial synthesis stage, and Step 3 represent the rapid accumulation stage. Different letters in bar chart (a, b, c, d) indicate significant differences (*p* ≤ 0.05) according to Waller-Duncan’s test.

In this study, known key genes for tanshinone synthesis, such as *SmCPS1* and *SmKSL1*, had low expression in the preparation stage, but showed a gradual increase in the expression in the initial synthesis stage and the rapid accumulation stage, consistent with the accumulation pattern of tanshinone [13,25]. Therefore, through the analysis of co-expression patterns, we found five candidate genes that are highly related to them and may be related to the synthesis of tanshinone [9]. The initial synthesis process of tanshinone in *S. miltiorrhiza* seedlings is displayed in Figure 7.

The DEGs number in the light red root vs. red root comparison (Q vs. R) was significantly higher than that in the white root vs. light red root comparison (B vs. Q). The tissue used for RNA-Seq analysis was the whole root. Therefore, the transcriptome data primarily reflected the whole root’s expression characteristics, including the root’s phloem and xylem and the root epidermis for synthesizing tanshinone. Previous studies explored the transcriptional characteristics of three tissue sites and confirmed that the structure of the periderm is closely related to tanshinone synthesis, which provides a reference for us to use the periderm to explore the information related to tanshinone synthesis and metabolism in the future [8,33]. The morphological structure difference between the white root and light red root (B vs. Q) was slight, but the color of the root epidermis and the content of tanshinone were significantly different. Some studies have also confirmed that there is a positive correlation between the color of the periderm and the content of tanshinone, which means more differential gene expression and metabolic activities [33,34]. Therefore, the analysis of DEGs and enrichment pathway will help to dig out the genes related to tanshinone biosynthesis and metabolism. KEGG analysis showed that the up-regulated differential genes were mainly involved in terpene synthesis, which confirmed that 25–30 days after germination was the critical period of tanshinone synthesis. In addition to terpene metabolic pathways, the plant hormone signal transduction pathway and the ABC transport pathway were significant enrichment pathways. Studies have shown that many exogenous hormones such as salicylic acid and gibberellin can induce the expression of essential enzyme genes in the tanshinone synthesis pathway, resulting in the change in tanshinone content in hairy roots [20,35,36,37,38]. When applied at the appropriate concentration, Brassinosteroid treatment significantly increased the essential oil content in peppermint (*Mentha piperita* L.) [39]. The interaction between exogenous cytokinin and ethylene significantly enhanced the geraniol ten hydroxylase gene (G10H), thus promoting the accumulation of alkaloids in *Catharanthus roseus* cell suspension [40]. In addition, plant ABC transporter genes have also been reported to play an essential role in regulating the transport and accumulation of secondary metabolites [41,42,43]. Previous studies have suggested that transporters may be involved in the synthesis and transport of tanshinone [44]. Therefore, we speculate that the differential genes of these pathways may be involved in the regulation of tanshinone synthesis, which is worthy of further attention and research.

Interestingly, the enrichment pathway of down-regulated genes in B vs. R had photosynthesis-antenna proteins, which are internal antenna light-harvesting proteins of oxygenic photosynthesis used to effectively absorb light energy [45,46]. This indicates that the roots near the ground surface within 15d may have many substances that may participate in photosynthetic reactions. By 30 days, the production of this substance related to the photosynthetic reaction is no longer significant.

The protein–protein interaction (PPI) network can intuitively show the relationship between protein interactions in organisms and can tap the core regulatory genes [47,48]. Several candidate proteins that may be involved in mediating tanshinone synthesis and regulation were screened by predicting the interaction network of differential proteins between white and red roots. Several vital enzymes downstream of the tanshinone synthesis pathway were found to be located in complex interaction networks, including *SmGGPPS* and three known CYP450s (CYP71D375, CYP71D373, and CYP71D411). Geranylgeranyl diphosphate (GGPP) synthase is a key enzyme that catalyzes the formation of diterpenoids such as tanshinone [49]. Three genes in the CYP71D subfamily play an important role in the tanshinone biosynthesis pathway: CYP71D373 and CYP71D375 catalyze hydroxylation at carbon-16 (C16) and 14, 16-ether (hetero) cyclization to form the D-ring, whereas CYP71D411 catalyzes upstream hydroxylation at C20 [12]. They may interact with two genes encoding photosynthetic ferredoxin (FD) and CCAAT binding transcription factor (CBF-B/NF-YA). It is known that many important plastid enzymes in cell processes rely on FD to provide electrons, including nitrogen assimilation, sulfur assimilation, amino acid synthesis, etc. [50]. Studies have shown that plant NF-Y plays a vital role in embryonic development, photosynthesis, root development, and stress response [51,52,53]. There were few studies on NF-Y transcription factors in secondary metabolism. At present, the NF-Y transcription factor has been proved to regulate the biosynthesis of flavonoids in tomato fruit by affecting the expression of the chalcone synthase gene (CHS1) [54]. Our results suggest that by interacting with critical enzymes, the NF-Y transcription factor may regulate tanshinone synthesis. In addition, multiple CYP450s were also predicted to participate in this interaction network, and most of them were up regulated in the red roots. This imply that these critical enzymes in the catalytic reaction form a protein complex to perform functions during the synthesis of tanshinone.

## 4. Materials and Methods

### 4.1. Plant Materials

Seeds of *S. miltiorrhiza* sprouted in a nutrition medium containing peat and vermiculite (1:1 by volume). Then, the seedlings were grown in a greenhouse at the State Key Laboratory of Crop Biology of Shandong Agricultural University at 25 °C with 16 h of light and a relative humidity of 60%. Seedling development was observed intermittently from the 15th day, and samples were taken every five days.

### 4.2. Morphological Characteristics

After 15 days of growth of all the transplanted seedlings, ten independent seedlings samples with the same growth were collected every five days. The roots were rinsed with running water, divided into three portions (three repetitions), and then dried to constant weight in an oven at 60 °C. The shoot and root biomass, the taproot’s diameter, and the root’s number and color were investigated.

### 4.3. UPLC Analysis of Tanshinone ⅡA Content

The content of tanshinones ⅡA was determined using an ultra-high-performance liquid chromatography (UPLC) analysis [55]. Ten independent seedlings samples with the same growth were collected every five days after 15 days of growth. The roots were rinsed with running water, divided into three portions (three repetitions), and then dried to a constant weight in an oven at 60 °C. Then, 0.1 g of powder was extracted with 10 mL of 80% methanol under ultrasound for 30 min at 30 °C. Hereafter, the sample was filtered through a 0.22 µm microporous membrane filter. The Waters UPLC system (Waters, MA, USA) was used to determine the content of tanshinone ⅡA. Chromatographic separations were performed using the Waters^®^ ACQUITYTM BEH C_18_ column (3.0 × 150 mm, 1.7  µm). Gradient elution was performed using a mobile phase of acetonitrile (A) H_2_O containing 0.05% phosphoric acid (B), included 20%A (0–5 min), 20–30% A (5–10 min), 30–60% A (10–15 min), 60–70% A (15–20 min), 70–80% A (20–25 min), and 80–100% A (25–30 min). The sample injection volume was set at 4 µL, and the flow rate was 0.5 mL/min. The tanshinone ⅡA was detected using 280 nm PDA wavelengths.

### 4.4. Microscopic Observation of Cell Morphology and Pigment Distribution of S. miltiorrhiza Root

Fresh roots samples grown for 20 d (white), 30 d (light red), and 60 d (red) of *S.miltiorrhiza* were cleaned with sterile deionized water. The thin root epidermis was quickly cut off by a double-sided blade and immediately placed in a little sterile deionized water at the center of the slide. Then, we gently covered the cover glass and drove out the bubbles. The samples were observed and photographed with an OLYMPUS SZX16 stereomicroscope.

Fresh hand-cut root sections of *S. miltiorrhiza* grown for 20 d, 30 d, and 60 d that were approximately 1cm were taken in glass scintillation vials and treated in phosphate-buffered saline (PBS) five times for 20 min each time. The samples were fixed in OsO_4_ overnight at 4 °C. Subsequently, the sample was rewashed with a PBS buffer five times and dehydrated in gradient ethanol (45%, 55%, 75% at 4 °C; 85%, 95%, and 100% exchanges at room temperature). The dehydrated sample was transferred to amyl acetate for treatment for one hour each time. The samples were critical point dried with liquid CO_2_. Then, the samples were stuck on the conductive carbon glue platform and transferred to the ion sputtering equipment for coating with 10 nm of gold. A scanning electron microscope was used for observation and image acquisition.

### 4.5. RNA Preparation, Library Preparation, and Sequencing

Taproot tissues of ten independent seedlings grown for 20 d, 30 d, and 60 d were randomly collected to construct a full-length cDNA library, which was named B (white root), Q (light red root), and R (red root). In total, 0.2 g of root samples in similar growth conditions were collected after removing the hair roots and were mixed afterward as one replicates. Three biological replicates were collected in each period. All the samples were frozen in liquid nitrogen and were stored at −80 °C for further experiments. The total RNAs from nine samples were extracted using the RNAprep Pure Plant Kit (Polysaccharides- and Polyphenolics-rich) (TIANGEN, Beijing, China) following the provided protocol and were treated with DNase Ⅰ to remove genomic DNA. The integrity and purity of the total RNAs were assessed with agarose gel electrophoresis.

The cDNA libraries were constructed using cDNA-PCR Sequencing Kit (SQK-PCS109) protocol provided by Oxford Nanopore Technologies (ONT). The final cDNA libraries were added to FLO-MIN109 flowcells and were ran on the PromethION platform at Biomarker Technology Company (Beijing, China). Guppy software in MinKNOW2.2 was used for the base recognition of raw data derived from Nanopore sequencing. Short reads, low-quality reads, and reads with connectors (minimum mean reads quality score = 6, minimum read length = 200 bp) were then filtered out, and ribosomal RNAs were removed. The FLNC transcript cluster with primers at both ends was identified and mapped to the reference genome of *Salvia miltiorrhiza* using the Genome Mapping and Alignment program (GMAP) and minimap2. Mapped reads were further collapsed with the cDNA_Cupcake package with min-coverage = 85% and min-identity = 90%. A 5′ difference was not considered when collapsing redundant transcripts.

### 4.6. Gene/Transcript Expression Level Quantification and Differential Expression Analysis

The full-length reads were mapped to the reference transcriptome sequence, and reads with a match quality more significant than five were further quantified. Expression levels were estimated by reads per gene/transcript per 10,000 reads mapped. CPM (counts per million) was used to measure the transcription or gene expression level [56]. DESeq2 was used for differential expression analysis. The method of Benjamini and Hochberg was used to control the error detection rate and thus adjust the resulting *p*-value. FoldChange ≥ 1.5 and *p*-value < 0.01 were selected as screening criteria for differential expression transcripts. FoldChange refers to the ratio of expression levels between two samples. *p*-value is the significance screening index of differentially expressed genes.

### 4.7. Gene Function Annotation and Enrichment Analysis

Gene function was annotated based on the following database: NR (NCBI non-redundant protein sequence), Pfam (protein family), KOG/COG/eggNOG (homologous cluster of proteins), Swiss-prot (a manually annotated and reviewed protein sequence database), KEGG (Kyoto Encyclopedia of Genes and Genomes), GO (genetic ontology). Gene body (GO) enrichment analysis of differentially expressed genes (DEGs) was achieved by GOseq R packages [57]. The KOBAS software tests the statistical enrichment of differentially expressed genes in the KEGG pathway [58,59].

### 4.8. Quantitative Real-Time Reverse Transcription PCR Analysis

The candidate DEGs were selected to carry out a tissue-specific expression analysis by qRT-PCR. The gene-specific primers were designed and assessed using the Primer Premier 5 software tool. Total RNA was reverse transcribed by the manufacturer’s recommendation using HiScript^®^ Ⅱ 1st Strand cDNA Synthesis Kit (Vazyme, Nanjing, China). A total of 2 µL of cDNA products were used as templates in a 20 µL qPCR reaction system. The Quantitative Real-time PCR reactions were performed on the QuantStudio™ 6 Flex Real-Time PCR System (Applied Biosystems, MA, USA) using a TransStart Top Green qPCR SuperMix (TransGen, Beijing, China). Each sample had three technical replicates. SmActin was used as an endogenous control to normalize the expression value. All the primers used are listed in Table S1. The relative quantitation was calculated by the Comparative CT (2^-−ΔΔCT^) method.

## 5. Conclusions

Tanshinone is synthesized in the root periderm of *Salvia miltiorrhiza* from a specific period. The tissue specificity and developmental specificity of tanshinone synthesis are the essential characteristics of *S. miltiorrhiza* as a medicinal material. Three key stages of seedling growth were preliminarily determined. A total of 4377 DEGs were screened by comparative transcriptome analysis, which were enriched in the terpenoid synthesis pathway, plant hormone signal transduction pathway, ABC transport pathway, and primary metabolism synthesis. Most genes involved in tanshinone synthesis were up regulated in the process of red root formation, which was consistent with the initial synthesis process. Five candidate genes that may participate in or regulate tanshinone synthesis were screened according to the co-expression pattern. Additionally, photosynthetic ferredoxin (FD), cytochrome P450 reductase (CPR), and CCAAT binding transcription factor (CBF) were predicted to interact with the known downstream essential enzyme genes directly, namely *SmGGPPS1*, *SmCYP71D375*, and *SmCYP71D373*. The above results are essential for analyzing tanshinone’s initial synthesis and regulation mechanism.

## Figures and Tables

**Table 1:** Phenotypic characteristics of *S.miltiorrhiza* seedlings within 60 days of growth (mean ± SD, *n* = 10).

Time (d)	Number of Blades	Fresh Weight of Leaf (g)	Fresh Weight of Root (g)	The Ratio of Root to Shoot	Taproot Diameter (mm)	Number of Roots	Color of Roots	Content of Tanshinone II A (mg/g)
15	2–3	0.198 ± 0.06 hg	0.032 ± 0.00 f	0.162	0.816 ± 0.14 f	1	white	——
20	3–4	0.301 ± 0.06 h	0.057 ± 0.01 e	0.189	1.251 ± 0.20 e	1	white	——
25	4–5	0.498 ± 0.13 g	0.122 ± 0.03 e	0.245	1.514 ± 0.33 e	1–2	white	——
30	4–5	0.643 ± 0.24 fg	0.180 ± 0.06 de	0.280	1.710 ± 0.32 de	1–3	light red	0.225 ± 0.02 d
35	4–6	0.904 ± 0.35 ef	0.295 ± 0.07 de	0.326	2.202 ± 0.39 cde	2–4	light red	0.477 ± 0.04 d
40	5–6	1.145 ± 0.44 de	0.471 ± 0.10 cd	0.411	2.530 ± 0.50 bcd	2–5	light red	1.232 ± 0.19 c
45	5–6	1.517 ± 0.50 d	0.661 ± 0.10 c	0.436	2.673 ± 0.54 bc	2–5	red	1.823 ± 0.14 c
50	5–6	1.974 ± 0.59 c	0.806 ± 0.10 c	0.408	2.994 ± 0.56 bc	3–5	red	2.989 ± 0.10 b
55	5–7	2.223 ± 0.68 c	1.070 ± 0.18 b	0.481	3.255 ± 0.64 b	3–6	red	3.237 ± 0.38 b
60	5–7	2.615 ± 0.707 b	1.426 ± 0.36 ab	0.545	3.613 ± 0.76 ab	4–7	red	3.449 ± 0.66 b
65	6–7	3.167 ± 0.88 a	1.788 ± 0.59 a	0.565	4.067 ± 0.71 a	5–7	red	4.208 ± 0.03 a

Different letters in each column indicate significant differences (*p* ≤ 0.05) according to Waller-Duncan’s test.

**Table 2:** Statistics of differentially expressed genes.

DEG Set	DEG Number	Up Regulated	Down Regulated
B1_B2_B3_vs_Q1_Q2_Q3 (BvsQ)	128	46	82
Q1_Q2_Q3_vs_R1_R2_R3 (QvsR)	3247	1365	1882
B1_B2_B3_vs_R1_R2_R3 (BvsR)	3672	1516	2156

## Data Availability

All data generated during this study are included in this published article and its supplementary information files, and the raw data used or analyzed during the current study are available from the corresponding author upon reasonable request.

## Supplementary Materials

The following supporting information can be downloaded at: https://www.mdpi.com/article/10.3390/ijms232113607/s1.
