# Supplementary material for: Spatiotemporal and Transcriptional Characterization on Tanshinone Initial Synthesis in Salvia miltiorrhiza Roots
Source: Int J Mol Sci. 2022 Nov 6;23(21):13607. doi: 10.3390/ijms232113607 (PMC9655840; doi:10.3390/ijms232113607)
Supplement: Supplementary file 1 [file ijms-23-13607-s001.zip › Figure S1-S8.pdf]

International Journal of
Molecular Sciences

MDPI

Article

Spatiotemporal and Transcriptional Characterization on Tanshinone Initial Synthesis in *Salvia miltiorrhiza* Roots

Caicai Lin [1,†], Lin Zhang [1,†], Xia Zhang [1], Xin Wang [1], Chaoyang Wang [1], Yufeng Zhang [1], Jianhua Wang [1,2], Xingfeng Li [1,2,*] and Zhenqiao Song [1,2,*]

1 Agronomy College, Shandong Agricultural University, Tai'an, Shandong 271018, China
2 State Key Laboratory of Crop Biology, Shandong Agricultural University, Tai'an, Shandong 271018, China
* Correspondence: lixf@sdau.edu.cn (X.L.); szq@sdau.edu.cn (Z.S.).
† These authors contributed equally to this work.

Supporting Information

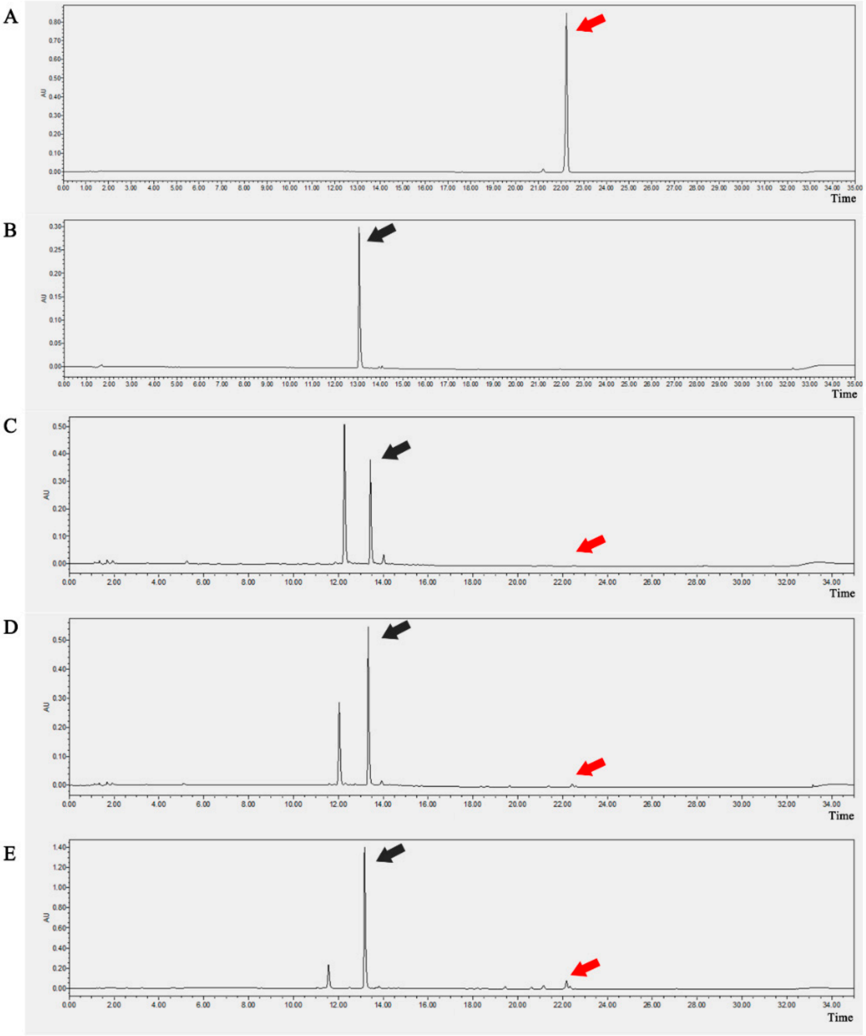

Figure S1. UPLC metabolite profiles of tanshinones IIA and salvianolic acid B in the root of *S. miltiorrhiza* plants during different growing periods. Red arrow: Tanshinone IIA; Black arrow: salvianolic acid B; A: The Tanshinone IIA standard; B: The salvianolic acid B standard; C: The roots grown for 20d; D: The roots grown for 30d; E: The roots grown for 60d (TIF).

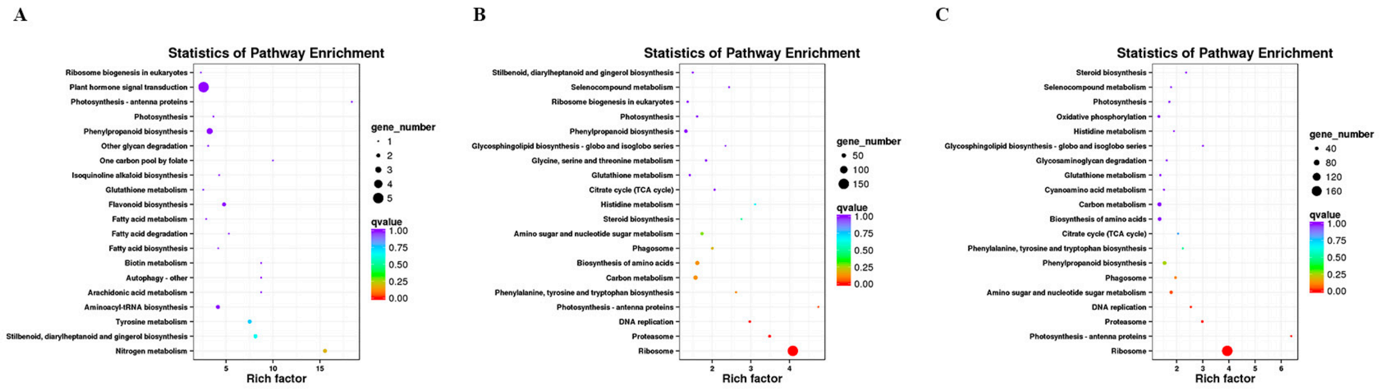

Figure S2. Scatter plot of KEGG pathway enrichment of down-regulated DEGs. A. B vs. Q; B. Q vs. R; C. B vs. R (TIF).

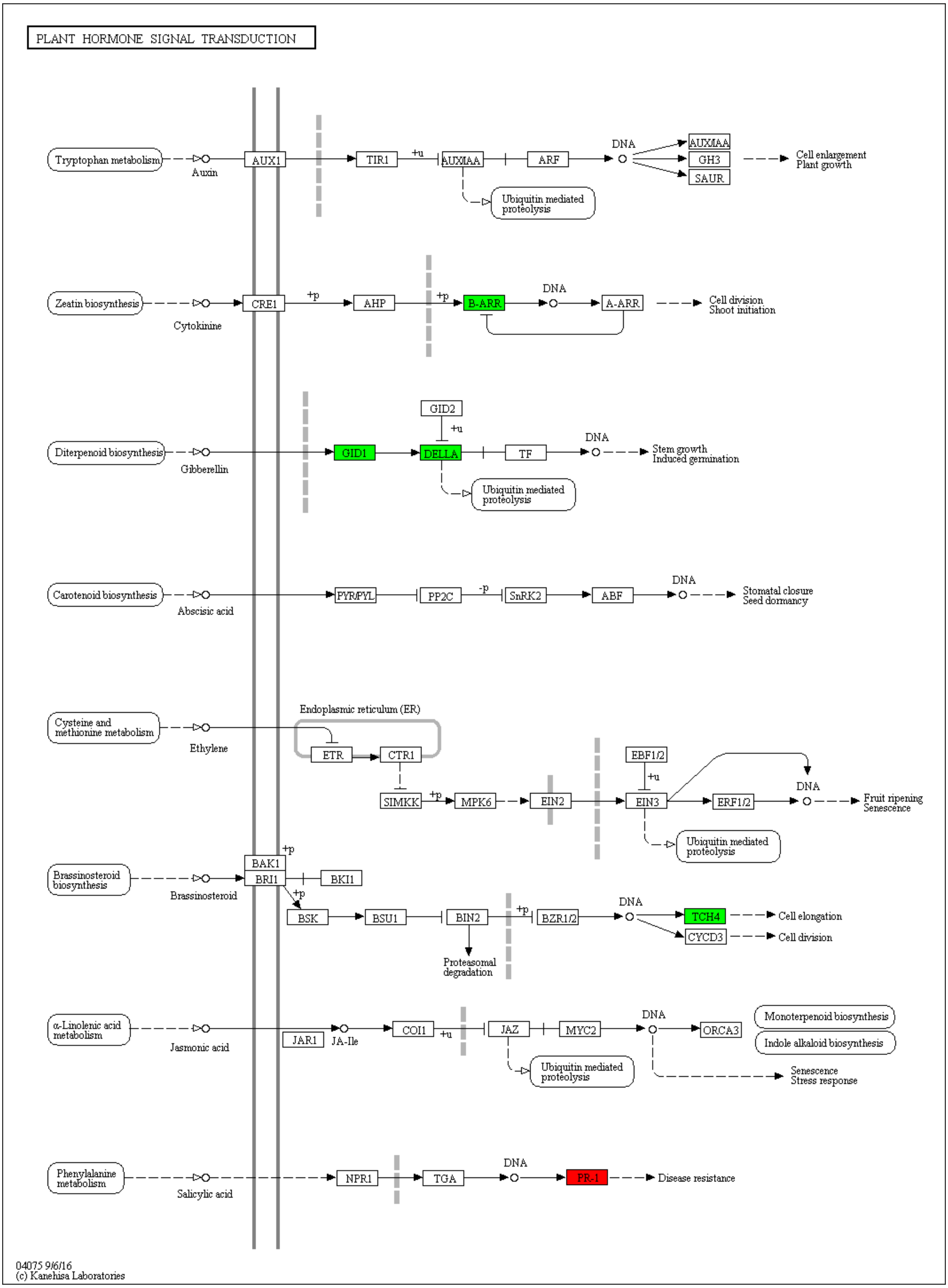

Figure S3. Graphs of plant hormone signal transduction pathway of DEGs in B vs. Q (TIF).

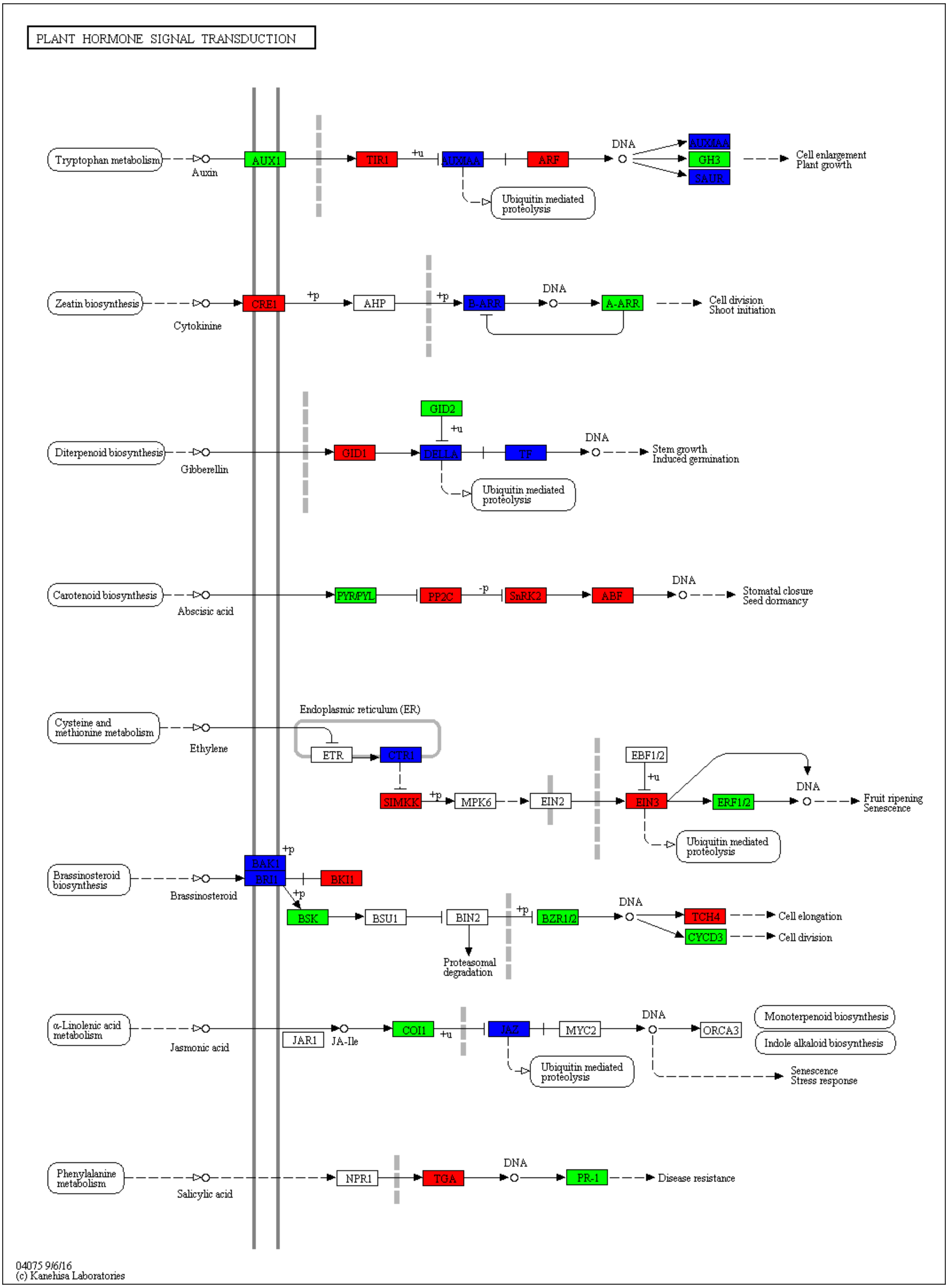

Figure S4. Graphs of plant hormone signal transduction pathway of DEGs in Q vs. R (TIF).

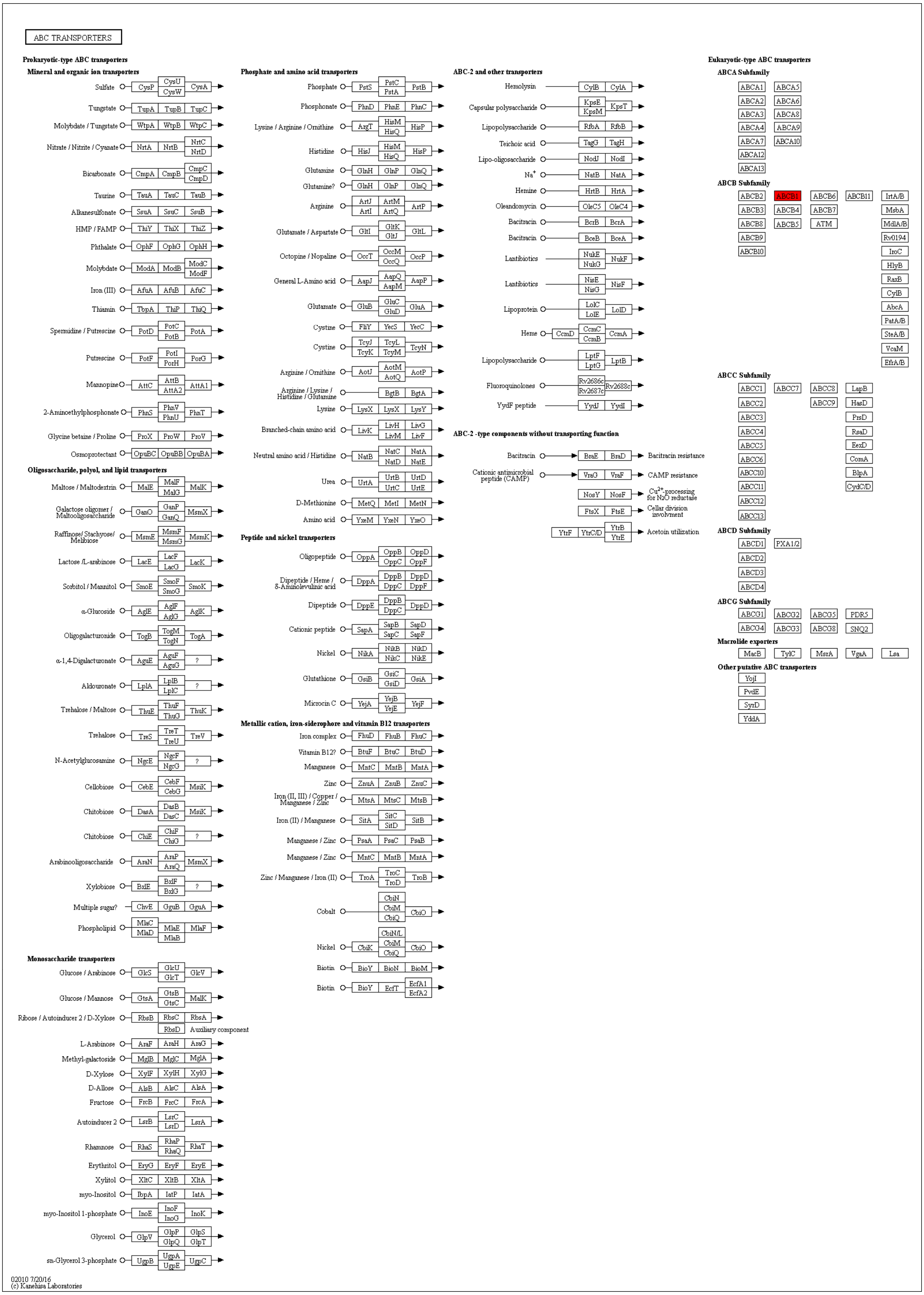

Figure S5. Graphs of ABC transporters transduction pathway of DEGs in B vs. Q (TIF).

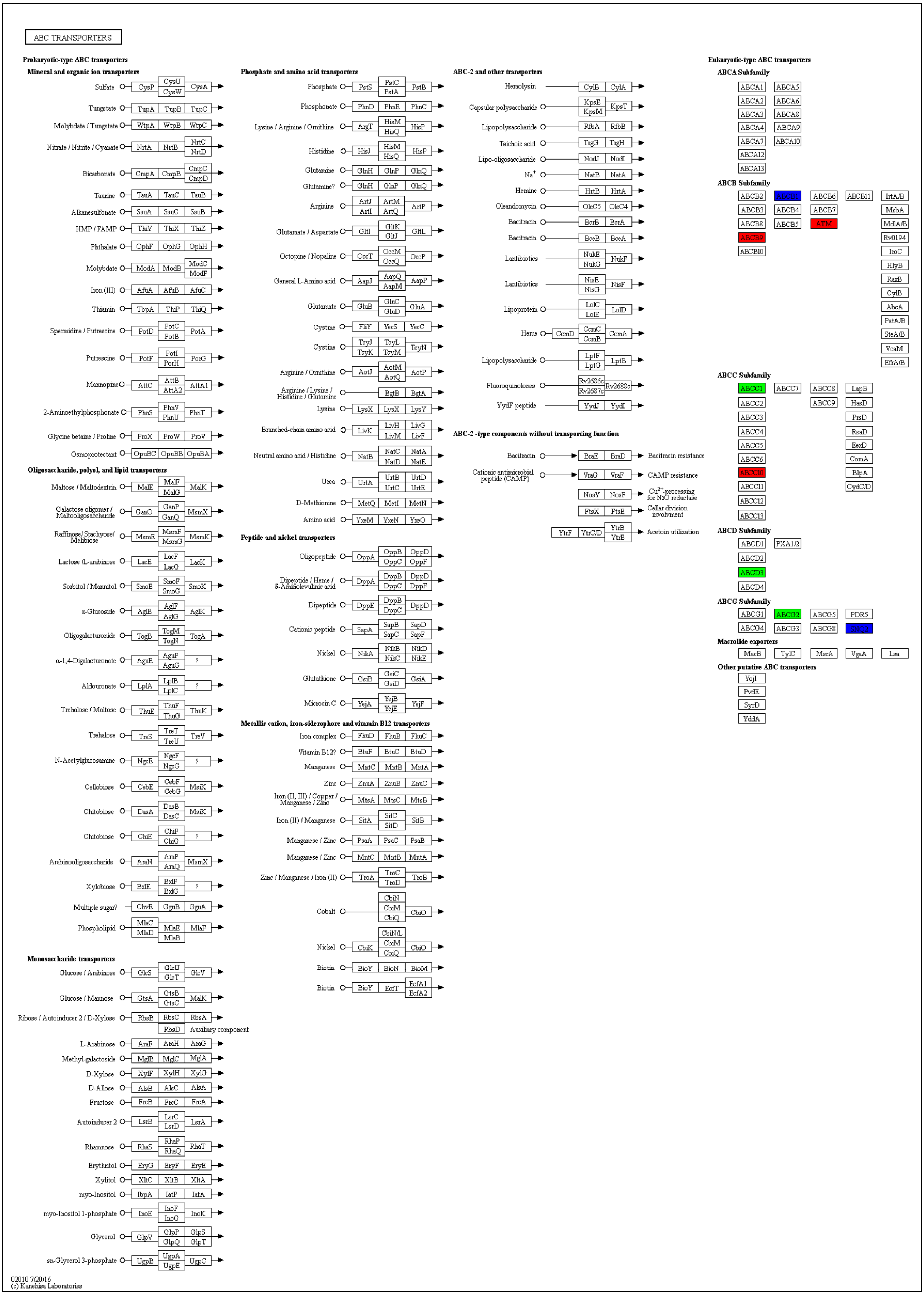

Figure S6. Graphs of ABC transporters pathway of DEGs in Q vs. R (TIF).

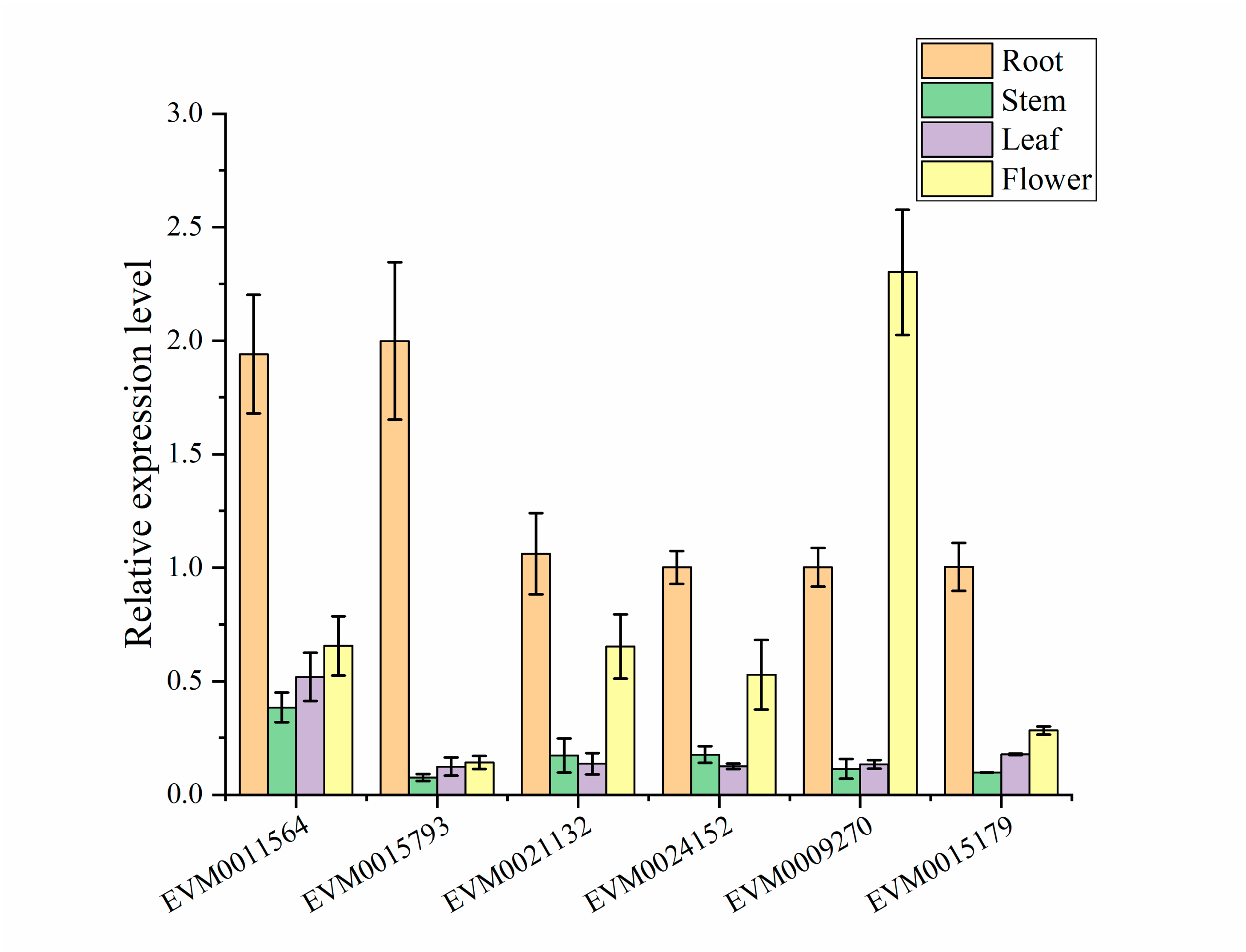

Figure S7. qRT-PCR analysis of DEGs selected by transcriptome data. Standard devation was represented by the error bars (TIF).

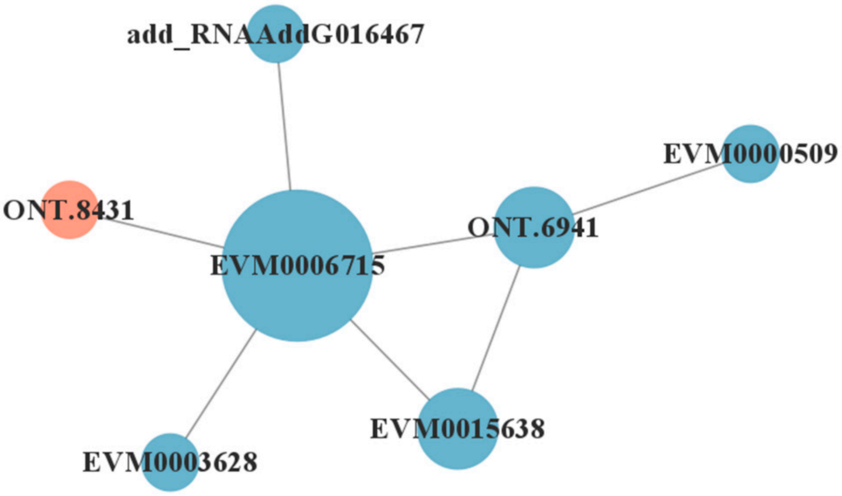

Figure S8. protein- protein interactions of DEGs in B vs. Q (TIF).

Table S1. Primers of DEGs for qRT-PCR. (XLSX).

Table S2. Statistics of data. (XLSX).

Table S3. Statistics of GO classification on DEGs. (XLSX).
